# Psychometric Properties of the Serbian Version of the Maslach Burnout Inventory-Human Services Survey: A Validation Study among Anesthesiologists from Belgrade Teaching Hospitals

**DOI:** 10.1155/2015/903597

**Published:** 2015-05-21

**Authors:** Bojana Matejić, Miodrag Milenović, Darija Kisić Tepavčević, Dušica Simić, Tatjana Pekmezović, Jody A. Worley

**Affiliations:** ^1^Institute of Social Medicine, Faculty of Medicine, University of Belgrade, 11000 Belgrade, Serbia; ^2^Center for Anesthesiology and Resuscitation, Emergency Center, Clinical Center of Serbia, 11000 Belgrade, Serbia; ^3^Institute of Epidemiology, Faculty of Medicine, University of Belgrade, 11000 Belgrade, Serbia; ^4^University Children's Hospital, Faculty of Medicine, University of Belgrade, 11000 Belgrade, Serbia; ^5^Department of Human Relations, University of Oklahoma, Oklahoma City, OK 73019, USA

## Abstract

We report findings from a validation study of the translated and culturally adapted Serbian version of Maslach Burnout Inventory-Human Services Survey (MBI-HSS), for a sample of anesthesiologists working in the tertiary healthcare. The results showed the sufficient overall reliability (Cronbach's *α* = 0.72) of the scores (items 1–22). The results of Bartlett's test of sphericity (*χ* ^2^ = 1983.75, df = 231, *p* < 0.001) and Kaiser-Meyer-Olkin measure of sampling adequacy (0.866) provided solid justification for factor analysis. In order to increase sensitivity of this questionnaire, we performed unfitted factor analysis model (eigenvalue greater than 1) which enabled us to extract the most suitable factor structure for our study instrument. The exploratory factor analysis model revealed five factors with eigenvalues greater than 1.0, explaining 62.0% of cumulative variance. Velicer's MAP test has supported five-factor model with the smallest average squared correlation of 0,184. This study indicated that Serbian version of the MBI-HSS is a reliable and valid instrument to measure burnout among a population of anesthesiologists. Results confirmed strong psychometric characteristics of the study instrument, with recommendations for interpretation of two new factors that may be unique to the Serbian version of the MBI-HSS.

## 1. Introduction

Burnout is defined as a syndrome of emotional exhaustion, depersonalization, and reduced personal accomplishment that is experienced in response to chronic job stressors that can occur in any kind of occupation, but mostly among human service professionals [1]. Numerous studies have confirmed that physicians and nurses experience very high levels of burnout, dissatisfaction, and work-related stress [2–4]. Burnout contributes to poor health outcomes of health care professionals, both in terms of physical illness and emotional problems [5]. This is followed by significant professional consequences (decreased work activity and demotivation, absence, impaired efficiency, impairment of relationships with other members of the health team, and high turnover intention rate) [6, 7], influencing the quality of care [8], patient satisfaction [9], and patient compliance [10].

The prevalence and severity of professional burnout have been reported across different medical specialties but most of the investigations explored the effects of work stress and burnout among intensive care unit professionals [11–13]. Previous studies have shown that anesthesiology is one of the most stressful specialties in medicine and can be associated with an increased risk of developing burnout syndrome among employees [14, 15]. The application of modern, invasive diagnostic, and therapeutic procedures and the introduction of increasingly complex medical technologies in the operating rooms and intensive care units significantly push the boundaries of patient survival but impose a more rigorous professional standard for employees. An interdisciplinary approach to treatment requires constant improvement of theoretical knowledge and skills and more complex work of anesthesiologists. The risk of developing occupational burnout is especially high for anesthesiologists responsible for the management and organization of the service [15, 16].

The most widely used instrument to measure burnout among healthcare professionals is the MBI-HSS (Maslach Burnout Inventory-Human Services Survey). A review of 34 burnout studies [17] in addition to results from other recent studies on the psychometric proprieties of MBI-HSS provides considerable evidence supporting the use of the Maslach Burnout Inventory-HSS as a useful measurement instrument for occupational burnout across a wide range of occupations, languages, and countries [18–20].

However, a commercial version of the MBI-HSS does not currently exist in Serbia. There are no known psychometric studies that have analyzed the factor structure of this instrument for a Serbian population. The use of the English version of the MBI-HSS with Serbian samples suggests the potential value of a Serbian language version of the MBI and demonstrates a desire to evaluate the burnout syndrome at work in the population of people whose primary language is Serbian. Moreover, the MBI has some known psychometric limitations that warrant caution in the use of the English version with populations for whom English is not the primary language [21]. This study is a first step toward the adaptation and validation of the MBI-HSS for use with Serbian speaking population.

There is a paucity of research on occupational burnout syndrome in the Serbian population. Existing studies of burnout have attempted to measure the construct with the English version of the instrument [22–26]. However, none of these studies present a psychometric evaluation of the instrument that was used to measure burnout.

Psychometric studies on the factor structure of the MBI-HSS or the original MBI—to which the MBI-HSS corresponds—began in the 1980s and the early 1990s with exploratory factor analysis (EFA) or principal components analysis (PCA). Confirmatory factor analysis (CFA) is now commonly used for testing hypothesized models of factorial validity after the fundamental factor structure is established.

A consistent finding with English translations is that the model fit for the original 22-item MBI-HSS (MBI-HSS-22) is poor (e.g., [27, 28]). In some studies, this problem was addressed by accepting correlations between the residual variances in the model and by allowing items to load across several factors in the tested model (e.g., [27]). There are theoretical arguments that question these types of solutions (see [28]). A more straightforward strategy is to remove the items that cause misfit in a hypothesized factor, but this approach makes sense only if the model is further validated. These issues with construct development in the English version are worth noting with considering the development of a Serbian translation of the instrument.

Shortened adaptations of the English version have been described and published with results from model testing procedures in the literature. The most frequent approach is to remove items 12 and 16. Several studies have observed acceptable-to-good fit for this 20-item MBI-HSS (MBI-HSS-20) (e.g., [29–31]). Item 12 was designed to measure personal accomplishment but was consistently observed with significant factor loadings on emotional exhaustion. Item 16 was designed to measure emotional exhaustion but was consistently observed with significant loadings on depersonalization.

It is interesting that the same problematic items were identified by different groups of researchers and that the shorter version is not substantially different from the MBI-HSS-22. Schaufeli and Enzmann [32] demonstrate measurement equivalence between the MBI-HSS-22 and MBI-HSS-20. The construct validity question has been addressed, and hypothesized models have been tested in non-English samples [29, 33, 34]. However, making a priori assumptions about the structure model for a Serbian version of the MBI-HSS is premature given the inconsistencies in agreement about the structure of the widely used English version of the instrument, namely, the identification of problematic items.

Despite known correlations among the three accepted dimensions of burnout in the English version of the instrument [35–37] only two factor-analytic studies have used oblique rotational procedures in the search for simple structure [38, 39]. Gold et al. [35] concluded that oblique rotational procedures (e.g., Promax, direct oblimin) are optimal when compared with orthogonal procedures (e.g., Varimax rotation).

Given the absence of previous psychometric studies on this instrument in Serbia and the existence of mixed results obtained with some adaptations to other languages [14], it is necessary to conduct an exploratory study of the factor structure of the MBI-HSS as a preliminary step to modeling the factorial structure in future studies using confirmatory factor analysis. Therefore, the objective of this study is to explore the factor structure of the MBI-HSS for the adaptation of the items conducted with no a priori assumptions of a multifactor model. As such, it will be possible to report findings from the translated and culturally adapted Serbian version of MBI-HSS (Maslach Burnout Inventory-Human Services Survey). The methodological approach begins by using exploratory factor analysis with oblique rotational structure for a sample of anesthesiologists working in the tertiary healthcare (teaching hospitals) in Belgrade.

## 2. Methods

### 2.1. Design and Sample

A cross-sectional survey was administered during the months of October and November 2013. All physician specialists of anesthesiology working in the hospitals at the tertiary level of health care in Belgrade (10 teaching hospitals) were eligible to participate in the study. Self-reported anonymous questionnaires were distributed by heads of the departments to 269 physicians. To increase interest in participation in the study, personal contact was established with the heads of the anesthesiology departments at each hospital. Excluded from participation were physicians who were on sick leave or holiday during the data collection period (approximately three weeks per institution), individuals with a discontinuity in the work of more than one year (prolonged studies abroad, prolonged illness, or multiple changes in the workplace over the past 5 years), and individuals who had previously been exposed to a short period of increased mental or physical trauma, independent of the professional environment. All prospective respondents were informed in writing that their participation was voluntary and that information provided would be treated confidentially. The participants provided their written informed consent to participate in this study. The ethics committee of Faculty of Medicine University of Belgrade, Serbia, approved the design of the study and consent procedure.

### 2.2. Measures

The level of burnout among anesthesiologists was assessed with the Serbian translation of the original 22-item version of the Maslach Burnout Inventory- Human Services Survey (MBI-HSS) [37]. This questionnaire assessed burnout across three dimensions. Emotional exhaustion (EE) was measured using nine items, depersonalization (DP) was measured using five items, and personal accomplishment (PA) was measured using eight items. Each of the 22 items asks respondents to describe their feelings on a 7-point Likert-type scale, ranging from never having those feelings to having those feelings a few times a week. Higher mean MBI subscale scores indicate higher feelings of emotional exhaustion, depersonalization, and/or personal accomplishment. Accordingly, high scores relating to emotional exhaustion and depersonalization correspond to a higher degree of burnout, but a high score for personal accomplishment corresponds to a lower degree of burnout on that dimension. Participants also completed a short questionnaire regarding their basic sociodemographic and work-related characteristics.

### 2.3. Translation and Cross-Cultural Adaptation of the Instrument

The authors obtained permission from Mind Garden, Inc., to translate the Maslach Burnout Inventory-Human Services Survey [37] into Serbian language and to use it for the period of one year. The translation and cultural adaptation of the instrument was performed according to the widely accepted principles of Good Practice Translation and Cultural Adaptation of Patient Reported Outcomes Measures [40], which included preparation, forward translation and reconciliation, back translation, harmonization, cognitive debriefing, and finalization. A bilingual translator independent of the study translated the original English version of the MBI-HSS into Serbian. This first Serbian version was subsequently back-translated and differences from the original wording were discussed by a panel of experts including anesthesiologists and English and Serbian language teachers. They used content correspondence as the main guideline to reach consensus on final item wording. The final version was tested in a pilot study that included 10 anesthesiologists, which confirmed a high level of item acceptance and comprehension. The same group of experts discussed the results of these tests. We assessed interrater reliability (IRR) and the overall percent agreement was 96.7%, signifying excellent agreement between respondents. This preliminary study confirmed that the Serbian language version was equivalent in content and meaning to the original American English version.

### 2.4. Data Analysis

Assessment of the psychometric properties of the MBI-HSS was conducted through the following parameters: (1) Acceptance is shown by the proportion of missing data at two levels: at the unit level and at the item level [41]. (2) Internal consistency reliabilities of the Serbian version of the MBI-HSS were assessed for multiple item scales using Cronbach's alpha coefficient, ranging from 0 to 1.0, the latter reflecting perfect reliability among scores for the sample. (3) Two tests assessed the suitability of data for structure detection: sample adequacy measured by the Kaiser-Meyer-Olkin (KMO) statistic predicts if data are likely to factor well, based on correlation and partial correlation, and Bartlett's test of sphericity was used to determine whether the correlations between the variables, examined simultaneously, do not differ significantly from zero. (4) In order to assess whether the allocation of items in the domain corresponds to their distribution in the original questionnaire (construct validity), an exploratory factor analysis (principal component analysis with Promax rotation) was conducted. A factor was considered important if its eigenvalue exceeded 1.0. In order to strengthen the argument for the number of factors, Velicer's MAP test was performed, focusing on the relative amounts of systematic and unsystematic variance that remain in a correlation matrix after extractions of increasing numbers of components [42]. Data collection and analysis were performed using IBM SPSS Statistics 19.

## 3. Results

A total of 205 anesthesiologists completed the survey, yielding a response rate of 76.2%. Missing data were assessed at the unit level and at the item level. The rate of item response was very high (98.53 to 100%), demonstrating excellent item response frequency on the MBI-HSS among Serbian anesthesiologists. Missing data at the item level were insignificant as the proportion of item response varied from 98.53 to 100%.

The basic characteristics of the study population are presented in Table 1. Over two-thirds (70.7%) of the current working anesthesiologists in the institutions of tertiary health care were females. The average age was 48.19 (SD = 8.31) years, ranging from 34 to 64 years old. All respondents were specialists of anesthesiology trained for four years after basic medical faculty. The average number of years of experience in the field of anesthesiology was 16.16 (SD = 8.98). More than one-third of respondents (36.6%) were in managerial positions for an average of 6.59 years (SD = 5.04). In addition, 31.2% reported that they had obtained additional academic achievements (M.S., Ph.D., or postdoctoral studies).

The mean scale scores and Cronbach's alpha values are presented in Table 2. The results showed that the overall reliability of the scores (items 1–22) was sufficient (Cronbach's *α* = 0.72), with the highest internal consistency value for the scale of emotional exhaustion (Cronbach's *α* = 0.91) and similar values for DP and PA.

The results of Bartlett's test of sphericity (*χ* ^2^ = 1983.75, df = 231, *p* < 0.001) and Kaiser-Meyer-Olkin measure of sampling adequacy (0.866) provided solid justification for factor analysis [43]. The data are approximately normally distributed, in terms of skewness and kurtosis *z* values, which are in the range of −1.96 to 1.96 [44]. In our validation study, the exploratory factor analysis model revealed five factors with eigenvalues greater than 1.0 (Table 3), explaining 62.0% of cumulative variance (EE: 32.07%, DP: 12.47%, PA: 7.22%, EPI: 5.58%, PPA: 4.64%). Velicer's MAP test has also confirmed five-factor model with the smallest average squared correlation of 0, 1844.

The majority of the items (81.8%) in the Serbian version of the MBI-HSS presented the highest loading weight in the expected domains based on the original development of the MBI as presented in Maslach and Jackson (1981) [37]. Specifically, all nine of the emotional exhaustion items loaded as expected.

Six of the eight personal accomplishment items loaded as expected, with item 9 and item 12 loading together as an independent factor.

Finally, four of the five items from the depersonalization subscale loaded together as expected. Items 11 and 22 did not load high on a single factor with other items expected to load as depersonalization. However, the highest loading for item 11 (0.47) was on the depersonalization factor. Item 22, on the other hand, loaded highest as a single-item factor (0.79).

An important difference compared to the original version was inclusion of two new factors, Energetic Positive Influence (EPI) and Feel Patients' Blame (FPB). The new factor named EPI was derived from item 9 “feel positively influencing people's lives” and item 12 “feel very energetic” of the original personal accomplishment subscale. The proposed FPB factor consists of a single item (“I feel patients blame me for their problems”; item 22), which was originally associated with the depersonalization subscale.

The mean scale scores and Cronbach's alpha reliability estimates of the new five-factor structure of the instrument are presented in Table 4.

In this solution, five factors correlate in the manner presented in Table 5. New factor EPI negatively correlates with three other scales (EE, DP, and FPB) and positively correlates with PA. Second new factor (FPB) positively correlates with EE and DP and negatively with PA and EPI.

## 4. Discussion

The Maslach Burnout Inventory (MBI) has been widely used in research for more than three decades and is recognized as the leading measure of burnout. The internal structure of the MBI has been a challenge for a number of exploratory and confirmatory factor-analytic studies. This study was designed to collect data from anesthesiologists in Belgrade, Serbia, teaching hospitals and test the psychometric properties of the Serbian version of the MBI-HSS.

The Serbian version of the MBI-HSS demonstrated similar reliability scores across the three original scales to its corresponding original English language form, found by Maslach and Jackson [37] among a sample of human service personnel. The internal consistency reliability estimates for scores on the DP (*α* = 0.73) and PA (*α* = 0.74) scales for the Serbian version of the MBI-HSS were similar to those found by authors of the instrument [37] (where DP = 0.77 and PA = 0.74, resp.) as well as the results from other validation studies [13, 45]. Results from a reliability generalization meta-analysis of coefficient alpha for the MBI presented evidence that the internal consistency reliability estimates for scores on the EE subscale are always highest compared to the other two subscales, and in most studies *α* ranges between 0.80 and 0.89. The higher coefficient alpha for the scores from the EE subscale in our results (*α* = 0.91) indicates an even higher level of coherence among the answers relating to this domain in our sample.

### 4.1. What Is the Factor Structure of the Serbian Version of the MBI-HSS?

The MBI is a generic type of questionnaire, which means it could be used in a variety of different settings. This fact directly introduces the difficulties in comparing burnout scores across these divergent subgroups. In this line, it is reasonable to explore the best fitting factors model, which comprehensively describes the specific cohorts. The majority of the validation studies tended to replicate the original MBI structure [19]. In order to increase sensitivity of this questionnaire, we decided to perform unfitted factor analysis model (eigenvalue greater than 1) which enabled us to extract the most suitable factor structure for the Serbian version of this study instrument. The results of this study support a five-factor model, with two new factors. The results from a majority of 45 studies included in a recent meta-analysis, both descriptive and empirical analysis, supported a three-factor model [19]. Yet, the authors of the meta-analysis [19] found from two- to five-factor models and reported modifications of the original instrument in nine studies. The reported heterogeneity may have contributed to difficulties in interpreting and comparing factor structure obtained in different studies. In our study, five main factors account for 62.0% of cumulative variance, explaining much more variance than the usual three-factor model. The new factor structure was also supported by the results of Velicer's MAP test, which is well known procedure for the increase of the objectivity in factors extraction.

This factor emotional exhaustion is commonly viewed as the basic manifestation and the critical component or key aspect of burnout [46]. In our study, this was the only factor that corresponded fully to the original, which is consistent with the findings in seven other studies [18]. Yet, this has not been the case in all previous studies. In Densten (2001) [47, 48] the EE items split across subscales such that items 6, 16, and 20 loaded together to form an independent factor referred to as* psychological strain*, with the remaining EE items clustering together as* somatic strain*. Also, contrary to the Poghosyan et al. study of nurses from eight countries (not including Serbia) [13], the two EE items related to “stress” and “strain” (items 6 and 16) did not load on DP in the current study. In our analysis, two items from the original 8-item structure of the PA subscale (item 9 and item 12) became part of the new factor (EPI). This is interesting because Maslach and Jackson [37] recommended removing item 12, and several other previous studies found improved model fit after removing item 12 (cf. [18], Table  3). Likewise, Vanheule et al. [18] found a best fitting model for a large sample of nurses in Belgium after eliminating items 12 and 16. The results of previous studies also reported that item 12 does not strongly fit into the PA subscale and tends to vary across subscales. Moreover, in some research item 12 did not load into any factor [49] or cross-loaded onto more than one factor [18, 50]. Densten revealed that item 9 did not belong to the PA [47, 48]. Our new subscale highlighted the importance of focused assessment of two dimensions particularly important in the work of anesthesiologists: to have strength and power “feel very energetic” and to positively influence the lives of their patients “feel positively influencing people's lives”. This is consistent with findings from previous studies with Serbian physicians [26] in which quality of life variables and emotional profiles were related with the problem of job stress and burnout. Furthermore, item 22 originally belonged to the DP subscale [21] but loaded as an independent factor in the current study. Worley et al. [19] reported that item 22 was problematic in several studies, because it either did not load into any factor or loaded on the EE subscale. In the current analysis, a high factor loading (0.79) supported the extraction of item 22 “I feel patients blame me for their problems” to a separate domain, which was not the case in other published studies. A possible explanation of this finding could be due to work-related characteristics in the field of anesthesiology. Namely, the anesthesiologists continuously deal with very challenging situations and decisions, literally influencing patients' lives. The original description of the depersonalization subscale is as “an unfeeling and impersonal response towards recipients of one's care or service” ([37], p. 101). Our view is that “depersonalization” does not accurately characterize the experience that a physician has when he or she believes a patient might think negatively about their professional role as an anesthesiologist or when a patient blames the service provider for the patient's problems (item 22). Rather, we believe this is in opposition to the construct and meaning of depersonalization. Therefore, the extraction of item 22 into a separate domain represents a focus on the responsibility and humanity of providers (anesthesiologists) who still care and respect the patients' opinions, not depersonalization, but perhaps also distinct from what González-Romá et al. [51] have labeled “identification.”

### 4.2. What Is the Nature of the Relationship among Factors on the Serbian Version of MBI-HSS?

There was a positive factor correlation between EE and DP (0.32). This is consistent with findings from 25 distinct samples of the English version, where the average correlation between these two factors in both EFA and CFA studies was essentially equivalent, indicating that shared variance between these two factors is 32%. Also, the factor correlation between EE and PA, and between DP and PA, was negative. This finding with the Serbian version of the MBI-HSS is also consistent with the finding in CFA studies reported in Worley et al. [19], providing support for the notion that PA is a distinct factor in the Serbian version as well. The negative relationship between EPI and the two burnout subscales (EE and DP) is consistent with the relationship between exhaustion and vigor on the burnout-engagement continuum that is discussed in other publications. The positive relationship between FPB “feel patient's blame” and the two burnout subscales (EE and DP) seems consistent with what González-Romá et al. [51] have labeled “identification.” These relationships between occupational burnout and work engagement have also been highlighted by other researchers [52].

## 5. Conclusion

Job burnout is influenced by several factors related with work role characteristics, organizational factors, personal characteristics of individual employees, and other contextual factors [53, 54]. The psychometric evaluation of the Serbian translation of the MBI-HSS represents an important step toward the development of a psychometrically sound measure of occupational burnout that can be adapted to the specific characteristics of the Serbian sociocultural context.

In conclusion, this study indicated that Serbian version of the Maslach Burnout Inventory (MBI) is a reliable and valid instrument to measure burnout among a population of anesthesiologists. The best fitted model for our sample was a 5-factor model. Results confirmed strong psychometric characteristics of the Maslach Burnout Inventory (MBI), which is observed in many other international studies, with recommendations for interpretation that may be unique to the Serbian version of the MBI-HSS.

## Figures and Tables

**Table 1:** Study population sociodemographic and work-related characteristics.

Variables	*N*	%
Gender		
Female	145	70.7
Male	60	29.3
Age (years)		
<35	7	3.4
35–55	149	72.7
≥56	49	23.9
Managerial position		
Yes	75	36.6
No	129	63.4
Academic achievement^*∗*^		
No	141	68.8
M.S.	41	20
Ph.D.	18	8.8
Postdoctoral studies	5	2.4

^*∗*^In Serbia, all academic degrees are awarded after a minimum of five years of medical university study and successful defense of a written thesis.

**Table 2:** Means, standard deviations, 95% confidence intervals, and internal consistency estimates on original subscales of the Maslach Burnout.

MBI subscales	Number of the items	Mean	Standard deviation	95% CI	Cronbach's *α*
EE	9	27.54	12.56	25.81–29.27	0.91
DP	5	5.94	5.45	4.18–6.01	0.73
PA	8	35.99	7.43	34.12–36.52	0.74

**Table 3:** Exploratory factor analysis of the Serbian version of the MBI-HSS.

Original MBI items	Factor 1	Factor 2	Factor 3	Factor 4	Factor 5
	EE	DP	PA	EPI	FPB
Emotional exhaustion (EE)					
(1) Feel emotionally drained from work	0.85^1,2^	−0.09	−0.05	−0.11	−0.11
(2) Feel used up at the end of the workday	0.85^1,2^	−0.11	−0.07	−0.01	−0.08
(3) Feel fatigued when getting up in the morning	0.79^1,2^	0.01	0.02	−0.13	0.03
(20) Feel like at the end of the rope	0.59^1,2^	0.21	0.12	0.31	0.01
(8) Feel burned out from work	0.88^1,2^	−0.06	−0.02	0.29	−0.10
(13) Feel frustrated by job	0.65^1,2^	0.06	−0.25	−0.08	0.04
(14) Feel working too hard on the job	0.82^1,2^	0.01	−0.01	0.34	0.01
(6) Working with people puts too much stress	0.52^1,2^	−0.05	0.06	−0.28	0.23
(16) Working with patients is a strain	0.61^1,2^	−0.03	0.10	−0.05	0.32

Personal accomplishment (PA)					
(4) Can easily understand patients' feelings	0.14	−0.41	0.52^1,2^	−0.12	0.13
(7) Deal effectively with the patients' problems	0.23	0.07	0.66^1,2^	−0.02	−0.40
(9) Feel positively influencing people's lives	0.01	−0.07	0.24	0.74^1,3^	0.15
(12) Feel very energetic	0.05	0.02	0.01	000.83^1,3^	−0.01
(17) Can easily create a relaxed atmosphere	−0.13	0.03	0.53^1,2^	−0.13	−0.46
(18) Feel exhilarated after working with patients	−0.16	−0.14	0.58^1,2^	0.0.0505	−0.18
(19) Have accomplished worthwhile things in job	0.08	0.14	0.61^1,2^	0.21	−0.06
(21) Deal with emotional problems calmly	−0.13	0.05	0.72^1,2^	0.08	0.16

Depersonalization (DP)					
(5) Treat patients as impersonal “objects”	0.13	0.83^1,2^	−0.06	0.01	−0.32
(10) Become more callous toward people	−0.10	0.83^1,2^	0.05	0.04	0.17
(11) Worry that job is hardening emotionally	0.30	0.47^1,2^	0.14	0.05	0.30
(15) Don't really care what happens to patients	−0.20	0.77^1,2^	0.04	−0.12	0.12
(22) Feel patients blame me for their problems	0.07	0.03	0.12	0.05	0.79^1,3^

Notes: ^1^highest factor loadings for each factor; ^2^factor loadings corresponding to the factors in the original version; ^3^factor loadings indicate highest loadings on factors other than the original ones.

**Table 4:** Means, standard deviations, 95% confidence intervals, and internal consistency estimates on five-factor structure of the Maslach Burnout.

MBI subscales	Number of the items	Mean	Standard deviation	95% CI	Cronbach's *α*
EE	9	27.54	12.56	25.81–29.27	0.91
DP	4	4.88	2.80	4.21–5.54	0.75
PA	6	28.39	5.61	27.62–29.1	0.72
EPI	2	7.59	3.09	7.17–8.02	0.67
FPB	1	1.06	0.44	0.86–1.26	—^a^

^a^A single-item measure produces no internal consistency reliability estimate.

**Table 5:** Component correlation matrix.

Component	EE	DP	PA	EPI^1^	FPB^2^
EE	0.91				
DP	0.317	0.75			
PA	−0.174	−0.328	0.72		
EPI	−0.353	−0.244	0.241	0.67	
FPB	0.431	0.378	−0.312	−0.345	—^a^

^1^Energetic Positive Influence (items 8 and 12).

^2^Feel Patients' Blame (item 22).

^a^A single-item measure produces no internal consistency reliability estimate.
